# Aim2Be mHealth intervention for children with overweight and obesity: study protocol for a randomized controlled trial

**DOI:** 10.1186/s13063-020-4080-2

**Published:** 2020-02-03

**Authors:** Louise C. Mâsse, Janae Vlaar, Janice Macdonald, Jennifer Bradbury, Tom Warshawski, E. Jean Buckler, Jill Hamilton, Josephine Ho, Annick Buchholz, Katherine M. Morrison, Geoff D. C. Ball

**Affiliations:** 1 BC Children’s Hospital Research Institute, School of Population and Public Health, University of British Columbia, F508 - 4480 Oak Street, Vancouver, BC V6H 3V4 Canada; 2 Childhood Obesity Foundation, Robert HN Ho Research Centre, 771A – 2635 Laurel Street, VGH Hospital Campus, Vancouver, BC V5 1M9 Canada; 3 Division of Endocrinology, Department of Paediatrics, University of Toronto, The Hospital for Sick Children, 555 University Avenue, Toronto, ON, M5G 1X8 Canada; 4 Cumming School of Medicine, Department of Pediatrics, University of Calgary, Calgary, AB T2N 4N1 Canada; 5 Children’s Hospital of Eastern Ontario Research Institute, 401 Smyth Road, Ottawa, ON K1H 8L1 Canada; 6 Department of Pediatrics, Centre for Metabolism, Obesity and Diabetes Research, 1280 Main Street W., HSC-3A, Hamilton, ON L8S 4K1, Canada; 7 Department of Pediatrics, Faculty of Medicine & Dentistry, University of Alberta, Edmonton, AB T6G 1C9 Canada

**Keywords:** Childhood obesity, Gamification, mHealth, Behavior change, Lifestyle intervention

## Abstract

**Background:**

The prevalence of overweight and obesity remains high in Canada, and the current standard for the treatment of childhood obesity is in-person, family-based, multidisciplinary interventions that target lifestyle behaviors (e.g., diet, physical activity, and sedentary behaviors). These programs are costly to operate, have limited success, and report recruitment and retention challenges. With recent advances in technology, mobile health or mHealth has been presented as a viable alternative to in-person interventions for behavior change, especially with teens.

**Purpose:**

The primary aim of this study is to test the efficacy of Aim2Be, a gamified app based on behavior change theory with health coaching to improve weight outcomes (i.e., decrease in standardized body mass index (zBMI)) and lifestyle behaviors (i.e., improve dietary quality, increase fruit and vegetable intake, reduce sugar-sweetened beverage intake, increase physical activity, and reduce screen time) among children 10- to 17-years old with overweight or obesity versus their peers randomized into a waitlist control condition. The secondary aims of this study are to 1) test whether supplementing the Aim2Be program with health coaching increases adherence and 2) examine the mediators and moderators of adherence to the Aim2Be intervention.

**Methods:**

We will employ a randomized controlled trial design and recruit 200 child and parent dyads to participate in the study (2019–2020). Participants will be recruited from Canadian pediatric weight management clinics and through online advertisements. Child participants must be between the ages of 10 and 17 years, have overweight or obesity, be able to read English at least at a grade 5 level, and have a mobile phone or home computer with internet access. Following baseline data collection, participants will be randomized into intervention and waitlist control groups. Intervention participants will receive access to Aim2Be, with access to health coaching. After having their data collected for 3 months, the control group will gain access to Aim2Be, with no access to health coaching. Participants will control their frequency and duration of app usage to promote autonomy.

**Discussion:**

Findings from this study will determine the efficacy of using Aim2Be in improving child weight outcomes and lifestyle behaviors and guide future mHealth interventions for pediatric weight management.

**Trial registration:**

ClinicalTrials.gov, NCT03651284. Registered 29 August 2018.

## Background

In Canada, approximately one-third of children aged 5–17 years have either overweight (19.8%) or obesity (11.7%) [1]. As children with overweight or obesity grow, they are more likely to develop a host of health issues including cardiovascular risk factors, Type 2 diabetes, asthma, sleep apnea, joint pain, and mental health issues [2–6]. Furthermore, numerous reports have shown that childhood obesity tracks into adulthood [7, 8], highlighting the need to develop efficacious and accessible lifestyle interventions for children and teens.

The causes of childhood obesity are complex and multifactorial [9]. Currently, the accepted standard of care for the management of childhood obesity is to provide in-person, family-based, multidisciplinary interventions that target lifestyle behaviors (e.g., diet, physical activity, and sedentary behaviors) and behavioral strategies at both the individual and familial levels [10, 11]. While such strategies are efficacious at decreasing weight or the body mass index (BMI) z-score [10, 11], their effects are modest, not sustained, and high attrition is a common problem [12–16]. In a German study that included 21,784 children attending a community weight management program, 76% and 92% of the participants dropped out after 6 and 24 months, respectively [14]. If these pediatric weight management interventions are going to achieve optimal impact for children and their families, the development and evaluation of more novel and engaging interventions that can meet the needs of these families and improve health outcomes are needed [17].

Mobile health (mHealth) technologies provide an opportunity to bring weight management interventions directly to individuals and to deliver such interventions on an ongoing basis throughout the day [18]. In children, mHealth interventions have predominantly targeted 12- to 15-year-olds, although some studies have included 15- to 19-year-olds [19]. Data on the efficacy of mHealth interventions for managing childhood obesity is still emerging [19, 20]. Importantly, mHealth interventions are ideally suited to 1) address some of the barriers associated with in-person lifestyle behavior modification interventions including the lack of enthusiasm for in-person meetings, lack of financial resources, inconvenient location, and lack of time [12, 15, 21, 22]; 2) emphasize the development of self-regulatory skills (e.g., goal setting, planning, and self-monitoring) that are hallmarks of effective lifestyle behavioral modification interventions [11], as specific app features can be designed to support self-regulatory processes; and 3) improve engagement by incorporating game-design elements without the context of what is traditionally considered a “game“ [23]. A review by Johnson et al. [24] identified that gamification can benefit health and wellbeing interventions, and that behavioral outcomes, in particular physical activity, may be the most ideal targets for gamification. With continued advances in wearable or transportable technologies (e.g., smart phones, smart watches, wireless activity trackers and other wireless devices) and the high prevalence of mobile phone use among children (5- to 15-year-olds spend an average of 15 h per week on a mobile phone [25, 26]), a need exists to harness the potential of mHealth lifestyle behavior modification interventions in the pediatric context, especially interventions targeting children 10 to 17 years of age.

To meet this need, an mHealth lifestyle behavior intervention—Aim2Be—was developed to promote healthy behaviors related to nutrition, physical activity, screen time, and sleep among children with overweight or obesity and their families. This paper describes our plan to assess the efficacy of Aim2Be in the context of a randomized controlled trial (RCT).

### Study aims and hypotheses

The primary aim of this RCT is to test the efficacy of Aim2Be with health coaching at improving weight outcomes and lifestyle behaviors among 10- to 17-year-old children compared to those randomized into a waitlist control condition. Children whose families receive the Aim2Be with health coaching are hypothesized to significantly decrease their age adjusted zBMI scores as well as significantly increase their moderate-vigorous physical activity, improve dietary behaviors (i.e., increase fruit and vegetable intake, improve dietary quality, and reduce sugar-sweetened beverage intake), and reduce screen time.

The secondary aims of the RCT are to 1) test whether supplementing the Aim2Be program with health coaching increases adherence to the intervention and 2) examine the mediators and moderators of adherence to the Aim2Be intervention. Given the exploratory nature of these aims, no hypotheses are stated.

## Methods/Design

### Study design

**Fig. 1:**
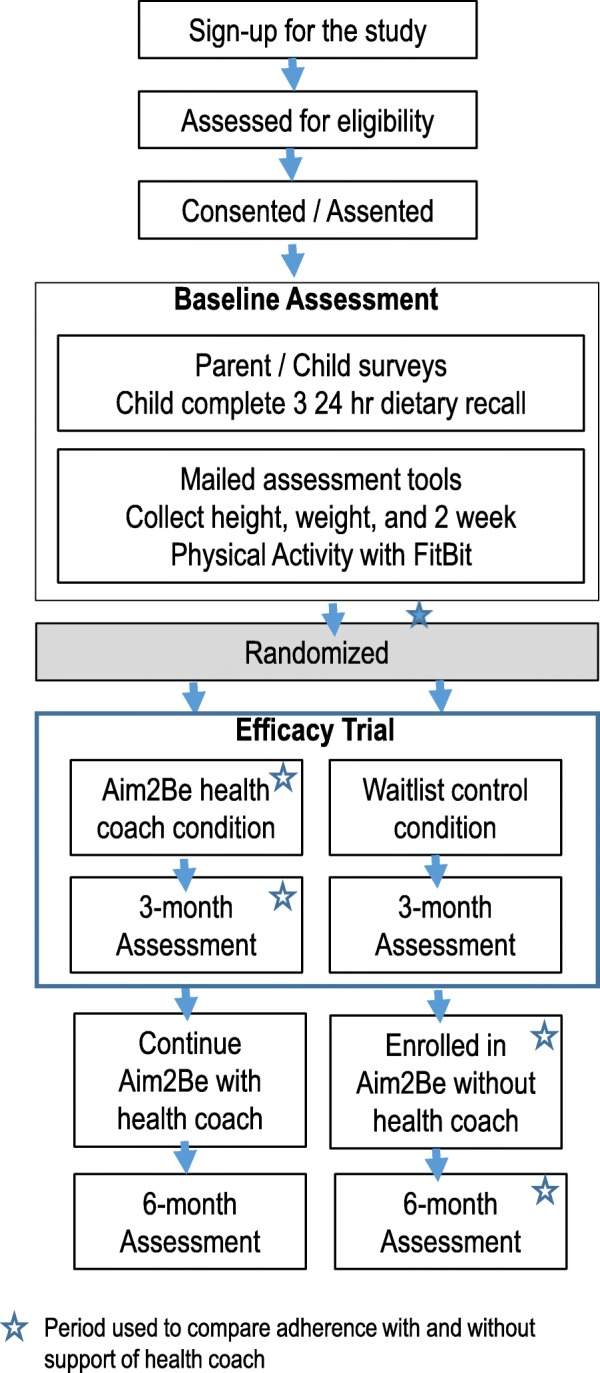
Study flow chart

Two hundred Canadian children who have overweight or obesity and one parent for each will participate in the trial, which is taking place in 2019–2020. Most participants will be recruited for a study funded in part by the Public Health Agency of Canada with matched funds from partners. A portion of the participants (*n* = 60) will be recruited as part of a study embedded within a Canadian Institutes of Health Research (CIHR) Team Grant in Bariatric Care (Team ABC 3) [27]. After consent and baseline measurements, families will be randomized into one of two conditions: 1) intervention or 2) waitlist control. Families randomized into the intervention condition will receive the Aim2Be app with health coaching (i.e., receive tailored messages from a health coach with the option of scheduled and unscheduled text support). All participants will receive a package containing a scale, measuring tape, activity tracker (Fitbit), and a brochure with current health recommendations. At 3 months, families randomized to the waitlist control conditions will move to the intervention for months 3 to 6, but they will not be supported by the health coach. Data collection will occur at baseline, 3 months, and 6 months, utilizing REDCap (Research Electronic Data Capture) [28, 29] hosted at BC Children’s Hospital Research Institute (BCCHRI). Staff involved in the data collection and analysis will not be involved in delivering the intervention. Ayogo Health Inc. and the Childhood Obesity Foundation (COF) will deliver the Aim2Be intervention. The BCCHRI/School of Population of Public Health (SPPH) at the University of British Columbia (UBC) will collect and analyze the data. The study flow chart is shown in Fig. 1.

A computer-generated (www.sealedenvelope.com) randomization schedule will be used to allocate participants in blocks of four, six, or eight participants with a randomization ratio of 1:1 [30]. The allocation schedule will be concealed in the randomization module of REDCap and only assigned after informed consent and baseline assessments have been obtained. Research team members will not enter or modify the allocation schedule, as it will be entirely computer generated. Participants will not be blinded to their allocation conditions because participants will know whether they receive the Aim2Be intervention immediately or in 3 months. Allocation assignment will be concealed to the researchers at the analysis stage.

### Study participants

#### Inclusion and exclusion criteria

Child participants must be 10- to 17-years old and literate in English, able to read at the grade 5 level or above, and have a mobile phone or a computer with Internet access at home. Child participants must have either overweight or obesity, based on the World Health Organization cut-offs for children and adolescents aged 5 to 19 (BMI > 85th percentile). Parent participants must be the caregiver with whom the child primarily lives and must be literate in English.

Children must not have a diagnosis of any musculoskeletal, cardiovascular, pulmonary, or orthopedic problems; disabilities precluding the participant from being physically active; any other physical condition that precludes the participant from being physically active; diagnosis of anorexia nervosa or bulimia nervosa; diagnosis of type 1 diabetes; dietary restrictions or special diets that limit a participant’s ability to eat a variety of foods; simultaneous participation in another physical activity, nutrition, or weight management study/program; use of medication, nutritional supplements, or herbal preparations to help lose weight; pregnancy; or a history of psychiatric problems or substance abuse that would interfere with adherence to the study protocol.

#### Recruitment

Participants will be recruited using two main methods: clinical site referral and social media (i.e., Facebook advertisements with a link to a study site). Seven clinical weight management programs across Canada are participating in this study and include BC Children’s Hospital (Vancouver, BC), Alberta Children’s Hospital (Calgary, AB), Stollery Children’s Hospital (Edmonton, AB), Misericordia Community Hospital (Edmonton, AB), McMaster Children’s Hospital (Hamilton, ON), The Hospital for Sick Children (Toronto, ON), and Children’s Hospital of Eastern Ontario (CHEO) (Ottawa, ON). Clinical sites will use a combination of mailing and emailing invitation and information packages as well as telephone contact. All potential participants will be provided an invitational package that describes the study and includes copies of the consent and assent forms, as well as a link to the study website.

For both groups of potential participants, interested individuals will be asked to complete a secure, online consent to contact form in REDCap and provide their contact information. Individuals who express interest in the study will be screened via telephone for eligibility.

#### Consent/assent process

All participants will provide informed consent or assent prior to taking part in this study. All parent participants, with the exception of parents of children 16 years of age or older recruited from CHEO, will be asked to sign a consent form consenting to their own and their child’s participation in the study. Parents of children 16 years of age or older recruited from CHEO will be asked to sign a consent form agreeing to their own participation in the study. All child participants, with the exception of those 16 years of age or older recruited from CHEO, will be asked to sign an assent form agreeing to their own participation in the study. Child participants 16 years of age or older recruited from CHEO will be asked to sign a consent form agreeing to their own participation in the study.

### Sample size calculation

Based on previously published data [31], the RCT will have 80% power at an alpha of .05 to detect a .5 decrease in BMI z-score when the sample size in each group is 60. The BMI z-score was used to calculate sample size because this is the most difficult variable to change and requires the largest sample size of all of the primary outcomes. Based on this and accounting for both missing data and attrition, we project needing at least 80 families in each condition. However, to be able to detect a 20% difference in adherence (e.g., secondary aims outcome) between the two intervention groups (odds ratio of 2.33) at an alpha of .05 with 80% power using a one-sided t-test, 77 families are needed in each group. As the waitlist control group will receive the intervention at 3 months, we require 100 participants enrolled in each group (this accounts for 15% attrition from baseline to 3-month and the approximately 8% of families who will not download the Aim2Be app). Power calculations were conducted in nQuery (Statsols, USA). Based on these computations, we aim to enroll 200 participants in the RCT.

### Development and description of Aim2Be

#### Aim2Be development

Aim2Be, which will be evaluated in this RCT, was built on the foundational knowledge learned in the first generation of the program called LiGHT (Living Green Healthy and Thrifty) and the second generation of the program, called Aim2Be version 1 (v1). LiGHT was an 11-module online program that integrated lifestyle behavior modification principles with environmental and financial concerns to address childhood obesity among 10- to 17-year-old children and their families [32]. Modest and nonsignificant improvements in readiness to change were initially observed for both the child and parents, likely due to ceiling effects on the measure and small sample size (*N* = 17 child-parent dyads). However, the qualitative feedback (*N* = 10 child-parent dyads) suggested that the LiGHT approach held promise. Specifically, both children and parents indicated that they related strongly to the foundational approach of the LiGHT intervention, meaning they liked having behaviors linked with health, environmental, and financial concerns. The evaluation also uncovered key areas that needed further improvement, including making the program more visually appealing, adding more interactivity, and creating a sense of community for the users.

The second generation of the intervention transitioned from a web-based intervention to a mobile app (iOs and Android) and was renamed Aim2Be. For those who do not have a smartphone, Aim2Be has remained accessible as a web-based platform via the internet. Aim2Be became a gamified app that supports children and their families to initiate sustainable behaviors in four primary areas: healthy eating, active living, reducing screen time, and healthy sleeping habits. It retained its focus on linking behaviors with health and living green, as well as adding emphasis on healthy body image and strong self-esteem. While Aim2Be retained some core elements from the LiGHT program, it 1) became strongly grounded in theories of behavior change (i.e., being family-based, supporting the development of self-regulatory skills at both the individual and familial levels, and enhancing self-efficacy through graded tasks) [10, 11, 19, 33]; 2) integrated principles of maintenance of health behaviors and as such it included self-regulatory processes that support intrinsic motivation [34–38]; 3) integrated gamification practices, which are designed to maximize both enjoyment and motivation [39]; 4) recognized the importance of body image and self-confidence in lifestyle management; 5) used the mHealth context to support self-regulatory processes [40]; 6) aligned with best clinical practices and guidelines in three areas (including a) the treatment of childhood obesity in Canada as it aligns with the curriculum of Canadian Weight Management programs (e.g., being family based, multi-behavioral, and focused on supporting skill development at the individual and familial levels); b) clinical guidelines for the management of childhood obesity as it integrates the central elements that need to be incorporated in these interventions (e.g., family focused, focused on improving lifestyle behaviors, skill based support) [41]; and c) Canadian health recommendations including the Canadian 24 Hour Movement Guidelines [42] and Canada’s Food Guide [43]); and 7) was designed specifically with and for children and their families (see details below).

Aim2Be was iteratively and incrementally developed. The first version of Aim2Be (v1) was field tested for 4.5 months among 301 teens between 14- and 17-years old and 315 parents. The quantitative evaluation revealed that teens who were moderately and/or highly engaged in the app (> 30 min of app usage) as compared to those with low engagement (≤ 30 min of app usage) significantly increased their motivation and self-efficacy to improve their dietary habits (i.e., limit sugar-sweetened beverages and increase intake of fruits and vegetables) and sedentary behaviors (i.e., limit screen time). At 4.5 months, children significantly increased their previous day’s intake of fruits and vegetables, decreased their consumption of 100% fruit juice, and reduced their screen time. In addition, parents who were more engaged with Aim2Be v1 reported significant improvements in the children’s dietary behaviors (i.e., increased intake of fruits and vegetables and decreased intake of sugar-sweetened beverages). Additionally, multiple rounds of qualitative evaluations were conducted among 36 teens/preteens and 24 parents. The qualitative evaluations included both focus groups and 2-week prototype testing, followed by semi-structured interviews. The results of these evaluations led to numerous improvements in Aim2Be including clarifying the overall purpose of Aim2Be, supplementing the tracking and check-in sections, adding more engaging features, and syncing the app with physical activity monitoring (i.e., Fitbit).

**Fig. 2:**
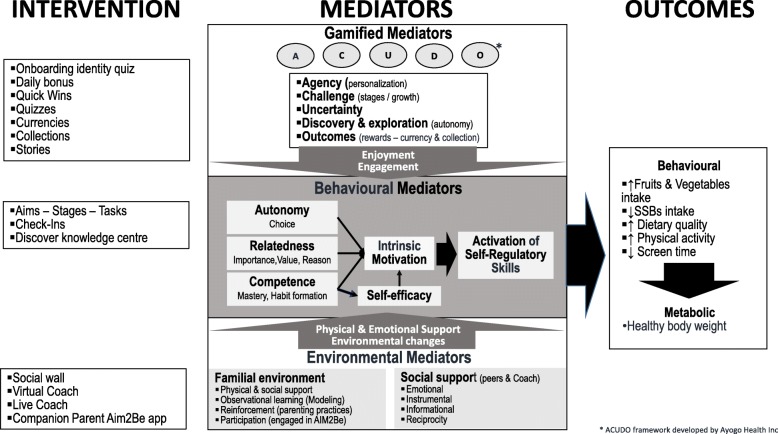
Conceptual framework of Aim2Be

The conceptual framework of Aim2Be is shown in Fig. 2. At its core, the behavior change techniques (BCT) incorporated in Aim2Be are rooted in 1) Social Cognitive Theory (SCT) [44]; 2) the Player Experience and Need Satisfaction (PENS) model, which is an extension of the Self Determination Theory model for the gamified context and incorporates enjoyment to support intrinsic motivation and the basic psychological needs that promote intrinsic motivation (autonomy, relatedness, and competence/self-efficacy) [39, 45]; and 3) the ACUDO framework [46]. The ACUDO framework, is a best practice framework developed by Ayogo Health Inc. [46] to promote engagement and enjoyment by: a) supporting Agency, b) incorporating Challenges, c) infusing Uncertainty, d) supporting self-Discovery and exploration, and e) adding fun Outcomes such as rewards. Specifically, Aim2Be includes strategies that target 1) gamified mediators of behavior change to ensure the experience is both enjoyable and engaging; 2) behavioral mediators that aim to activate self-regulatory skills and support the development of intrinsic motivation and increase self-efficacy as a way to support behavior change and ultimately support healthy body weights; and 3) environmental mediators because it is recognized that behavior change needs to be physically and emotionally supported in the familial environment and that social support from both peers and/or coach is also important to changing health behaviors (see Fig. 2).

#### Aim2Be features

Aim2Be is not a prescriptive app, but instead, it utilizes strategies to activate self-regulatory skills through a self-guided journey of self-discovery of current health behaviors and autonomy to select which health behaviors the user wants to work on. Strategies employed by the various app features are grounded in Michie’s behavior change taxonomy, which specifies the “active ingredients“ of behavior change interventions [47]. Children will be subdivided into two versions of the app based on their age: teens (13–17 years old) and preteens (10–12 years old) and will be able to select which aims they wish to address. Once an aim is selected, teens start a stage, and preteens start the aim as a whole, and then are provided tasks to help them set incremental goals, plan, and self-monitor their behaviors. In the application environment, the users progress along their journey by completing games, tasks, and activities including quizzes. The user receives currency that they can use to unlock collectibles and “choose your own adventure” stories. Users are provided with tools within the app to further support their journey including Check-Ins, Quick Wins, Articles, Quizzes, and a moderated Social Wall with daily topics to help engage users. Features that are incorporated in the preteen, teen, and parent apps, as well as the BCT [47] targeted by these features, are summarized in Table 1.

**Table 1:** Summary of Aim2Be© features and behavior change techniques

App feature	Description	Behavior change taxonomy [47]
Gamification components		
Profile/Onboarding *PT, T*	After a user creates an Aim2Be account, the user can play the game, “What animal are you?” The game involves answering a variety of questions about the user’s preferences, with various question formats. For example, users might be asked whether they prefer spending time with people or spending time alone. At the end of the game, the user is presented with an animal avatar recommendation (e.g., lynx, tiger, panda, etc.) along with adjectives describing that identify profile (e.g., “wild, determined, unafraid, intelligent, & habitual”). The users are given the option to select a different animal avatar, if they wish, to represent them in the app and the option to further customize their animal avatar, by selecting clothes and accessories.	13.4 Valued self-identity
Daily Bonus *PT, T*	Each time a user opens the app, the user is invited to break a piñata from which extra currency can be gained.	10.2 Material reward (behavior)
Quick Wins *PT, T, P*	Each day, a new Quick Wins appear on a side bar of the users home page. Quick Wins are quick, simple, and straightforward tasks that prompt the user to do something positive for their health that day (e.g., “Stand up. Shake it out. Stretch. Reach up to the sky, then to the floor.”) or to encourage them to explore a new aspect of the app (e.g., “Check out Aimbot for help.”). Users earn currency dollars for each day they complete a Quick Win.	4.1 Instruction on how to perform a behavior
		8.1 Behavioral practice/rehearsal
		8.2 Behavior substitution
		8.3 Habit formation
		8.4 Habit reversal
		8.7 Graded tasks
		10.4 Social reward
Quizzes *PT, T*	Users can complete short quizzes within the app. Whether the user selects the correct or incorrect answer, a pop up appears after each question with an explanation of the correct answer. Users earn currency diamonds for each quiz they complete.	2.2 Feedback on behavior
		4.2 Information about antecedents
		5.1 Information about health consequences
		5.3 Information about social and environmental consequences
		5.6 Information about emotional consequences
		6.2 Social comparison
Currencies *PT, T*	Users collect currency dollars and currency diamonds for engaging with different components of the app. These currency dollars and diamonds can be used to purchase collectibles and stories, respectively.	10.2 Material reward (behavior)
Collections *PT, T*	Collections include a variety of virtual items (e.g., skateboard, phone, lipstick, sneakers, etc.), divided by level, that a user can purchase with a certain number of currency dollars.	10.2 Material reward (behavior)
Stories *PT, T*	Users can use their currency diamonds to purchase interactive and/or engaging stories. Each story is divided into multiple chapters that are “unlocked” as a user reaches a new level.	10.2 Material reward (behavior)
	Stories present users with a fictional situation and character who is faced with a series of decisions. Users guide the fictional character through the situation by making decisions in a choose-your-own-adventure format. A recent study suggests that when individuals “lose themselves” in the world of a fictional character, they change their own behavior and thoughts to match that of the character. This phenomenon has been termed experience-taking [48]. Stories are designed such that users will empathize and identify with the fictional characters, allowing them to have experiences that they may not have yet had the chance to encounter in their regular lives. This allows them to shift their self-perception to include their “new experiences” and begin to identify themselves as the type of person who chooses healthy patterns of action.	9.3 Comparative imagining of future outcomes
		16.1 Imaginary punishment
		16.2 Imaginary reward
		6.1 Demonstration of behaviors (symbolic role modeling)
Behavioral Components		
Aim *PT, T, P*	Aims are high-level goals that users can choose to pursue, such as “Be sugar smart.” Users may pursue one aim at a time or may pursue up to three aims concurrently.	1.3 Goal setting (outcome)
		1.4 Action planning
	Once a user selects an aim to pursue, the user is first asked to indicate why they selected the aim, by completing the sentence, “I want this because....” Next, the user is asked to indicate on a Likert scale the importance of the aim, and their confidence in being able to achieve it. Following this, the user is asked to note any obstacles they might face in working toward the aim. Lastly, the user is asked to adopt a time frame for achieving their aim; they can either accept a recommended time frame or create a custom time frame.	1.6 Discrepancy between current behavior and goal
		1.9 Commitment
		13.5 Identity associated with changed behavior
	Users also have the option to request support from their parent or guardian or from a peer. Users can write their specific needs related to tasks and aims and send these to their desired recipient in email form through the application.	
	Users are given daily tasks related to the aim they set and are congratulated and awarded currency as they complete tasks and stages. When an aim is completed (all the tasks have been accomplished), the aim is moved to the “Completed aims” folder.	
Stage *T, P*	Aims consist of several stages that break the high level goal into more focused components. For example, the aim “Be sugar smart” includes the stages “Be sugar aware,” “Swap some sugar out,” and “Find a balance.” The Preteen app does not include stages, only aims and tasks.	1.1 Goal setting (behavior)
		1.5 Review behavior goal(s)
		1.7 Review outcome goal(s)
Task *PT, T, P*	Stages consist of a number of tasks that break the aim and stage down into small, achievable tasks. For example, the stage “Swap some sugar out” contains tasks such as replacing sugary drinks with water, reading a specific article in the app, reading labels to identify sugary foods in the home, and making a list of snacks eaten and circling those that are high in sugar.	1.2 Problem solving
		1.4 Action planning
		4.1 Instruction on how to perform a behavior
		4.2 Information about antecedents
		7.1 Prompt/cues
		8.1 Behavioral practice/rehearsal
		8.2 Behavior substitution
		8.3 Habit formation
		8.4 Habit reversal
		8.7 Graded tasks
		10.4 Social reward
		13.2 Framing/reframing
Check-In *PT, T, P*	At any time, users can access and update their health behavior status. The Check-In area provides a graph-formatted overview of how they are doing in terms of six self-rated health behaviors: sugary drinks, veggies and fruit, activity, family meals, screen time, and sleep. Each behavior has a short description of the health behavior recommendation, and users are asked to rate how they think they’re doing on a scale from poorly to not very well to okay to well to great.	2.2 Feedback on behaviors
		2.3 Self-monitoring of behaviors
		2.4 Self-monitoring of outcome(s) of behavior
Discover *PT, T, P*	Articles are an in-app resource for users who wish to learn more about particular topics. The Discover Center allows users to explore educational content at their own pace. Content is organized by health behavior topic and takes the form of relevant health articles that users can consume quickly and refer back to over time. Users are prompted to provide a short written response to a question related to the article content (e.g., “What is your biggest challenge in sticking to a healthy breakfast routine?”). They are provided with currency when they respond. Users are also prompted to indicate the value they derived from articles; user ratings are combined to indicate popular articles.	4.1 Instruction on how to perform a behavior
		4.2 Information about antecedents
		5.1 Information about health consequences
		5.3 Information about social and environmental consequences
		5.6 Information about emotional consequences
		7.1 Prompts/cues
Environmental components		
Social Wall *PT, T, P*	The Social Wall offers a safe social space within the application where users can come together and share thoughts, feelings, successes, and challenges around their healthy lifestyle goals. Prompts guide behavior change through feeling connected, listening, having a voice, and shifting norms. Humans are social creatures and our social networks play a huge part in our lives. Social networks have been shown to influence health outcomes like weight and medication adherence. This is especially true if the health issue has stigma surrounding it. When uncertainty is high, users are more influenced by their peers. Offering a safe social space where users can come together around common health goals is therefore considered imperative for long-term success. Self-efficacy, the most important influence on behavior change, is boosted by having access to a social space that is promoting healthy behaviors. The social wall will be moderated by the health coach and has some automated moderation built in to hold posts with inappropriate language. The moderator can review and approve the held posts; otherwise, posts go live immediately.	3.1 Social support (unspecified)
		3.2 Social support (practical)
		3.3 Social support (emotional)
		6.1 Demonstration of behaviors
		6.2 Social comparison
		13.1 Identification of self as role model
Virtual Coach *PT, T, P*	The Virtual Coach, Aimbot, is a digital bot with a range of pre-programmed prompts, questions, and answers designed to guide users in the app with behavior change in an empathetic way. All app users can access Aimbot. The content is based on queries that arose during the pilot.	3.2 Social support (practical)
Health coach *PT, T, P*	The health coach, trained in motivational interviewing, provides support to app users through an in-app coaching interface in which the Coach and user can have one-on-one messaging conversations to provide more tailored and more personal health behavior guidance.	2.2 Feedback on behavior
		3.1 Social support (unspecified)
		3.2 Social support (practical)
		3.3 Social support (emotional)
		10.4 Social reward
Parent Companion App *P*	The Parent Aim2Be Companion App complements the child’s Aim2Be app. It includes many of the same components (Quick Wins, Aims, Stages, Tasks, Health Behavior Check-Ins, Articles, Social Wall, Virtual Coach, health coach) and similar nutrition, activity, screen time, and sleep related content, but focuses primarily on how parents can support their children in making healthy behavior changes. The child and parent apps are not connected. The parent app is not gamified.	3.1 Social support (unspecified)
		3.2 Social support (practical)
		3.3 Social support (emotional)
		12.1 Restructuring physical and social environment

PT = Preteens; T = Teens, P=Parents

### Study conditions

Intervention condition participants will have access to the full version of the Aim2Be app (as described in Table 1) for 6 months. In addition, participants (teens and parents) will have the option to access a health coach who can support them in health behavior change using motivational interviewing techniques. The health coach has training in motivational interviewing and expertise in lifestyle management and working with families. The health coach will communicate with participants through the in-app text feature, and participants have the option to schedule a live text session if they wish to do so. The health coach will send an initial contact to all participants and will send follow-up supportive messages. The exact messaging and frequency of messaging that the health coach will provide to participants cannot be predicted, as this coaching will be individually tailored to the questions and concerns participants raise with the health coach, and the frequency with which they reach out to the health coach. This is in line with the autonomy support model featured throughout Aim2Be, where participants can choose the health behaviors on which they wish to focus.

Waitlist control condition participants will be put on a waitlist for 3 months during which time they receive a brochure with Canadian health recommendations about physical activity, diet, screen time, and sleep habits (this represents the duration in which they are part of the efficacy trial—see Fig. 1). Once their 3-month assessment is complete, they will have access to Aim2Be app, with no health coach, for the following 3 months. Participants will have access to all features of the Aim2Be app except the health coach and will be assessed 3 months after they were given access to the Aim2App with no health coach. This 3-month period will be compared to the 3-month period of the intervention condition to determine differences in adherence in Aim2Be with and without health coach support.

### Study data

**Fig. 3:**
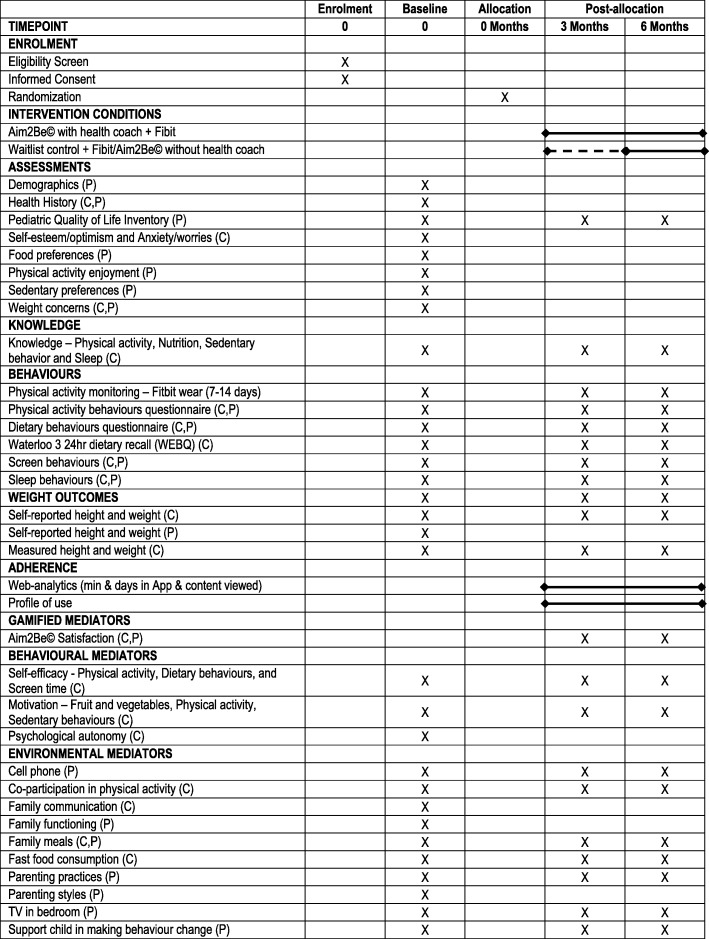
SPIRIT Figure

Data collection occurs at baseline, 3 months, and 6 months, and at each time point, the participating parent and their teen will complete an online survey administered using REDCap. The teen will also complete three 24-h dietary recalls using the Waterloo Eating Behavior Questionnaire (WEBQ), and parents will measure their teen’s height and weight following the protocol and tools provided by the research team. Finally, the study team will extract 14 days of Fitbit Flex 2 (Fitbit Inc., San Francisco, CA) wear for each teen at each assessment time point using Fitabase (Small Steps Lab LLC, San Diego, CA, USA), an online platform designed specifically for research using Fitbits. Participants will receive their Fitbit at baseline. *See* Fig. 3 for the SPIRIT Fig [49, 50]. to see when data collection occurs and for a detailed list of data that are collected to address the primary and secondary aims of this study. The complete SPIRIT checklist can be found in Additional files 1 and 2.

### Primary outcomes

#### Change in weight outcomes

Height and weight will be measured by parents using the Centers for Disease Control and Prevention home protocol [51] and will be used to calculate Body Mass Index (BMI) kg/m^2^; this is a valid and reliable method to assess BMI in children [52]. Each participant will be mailed a digital scale (Active Era) and tape measure (HDX) so that all measurements are taken using the same instruments. This procedure has been validated to assess height and weight at home among adolescents and found to provide valid assessment [52]. Teen BMI z-scores will be computed using a Stata macro developed by the World Health Organization, whereby a BMI z-score > 1 and ≤ 2 Standard Deviation = overweight and > 2 Standard Deviation = obese) [53].

Change in physical activity behaviors will be assessed using a Fitbit Flex 2, which can be immersed in water [54] (Fitbit Inc., San Francisco, CA), and with a questionnaire. Each participant will be mailed their Fitbit Flex 2 at baseline. From the Fitbit Flex 2, total steps per day for a period of 14 days will be extracted using the Fitabase platform. The child physical activity questions were modelled after the International Physical Activity and the Environment Network questions [55], and they ask about participation in physical education at school in the past week, involvement in team sports, and number of days of moderate-vigorous physical activity in the previous week.

Change in dietary habits will be measured using the WEBQ, a 24-h dietary recall web-questionnaire developed by the University of Waterloo, which has been validated in children [56], and with a questionnaire. Three 24-h dietary recall WEBQs (including 1 weekend day) will be analyzed using the Food Processor Software (ESHA Research, Salem, OR) using the 2015 Canadian Nutrient data file. An index of diet quality using the Healthy Eating Index adapted for Canadian food recommendations will be computed. The index computes a score from 0 to 100, where ≤ 50 is categorized as poor diet, 50–80 as needing improvement, and ≥ 80 as being good [57, 58]. In addition, seven dietary questions were included in the child’s questionnaire, and these questions were taken from the 2016 Canada Community Health Survey [59], with some questions originating from the Behavioral Risk Factors Surveillance System [60] and Centers for Disease Control and Prevention National Youth Physical Activity and Nutrition Study [61]. Questions asked about previous week and previous day consumption of fruits and vegetables, 100% fruit juices, and sugar-sweetened beverages.

Change in sedentary behaviors will be measured with an adapted version of French et al.’s assessment of screen time which has been found to be sensitive to intervention-mediated change [62]. Two questions ask the amount of time children spend in front of screen in their free time on weekdays and weekend day.

### Secondary outcomes

Adherence will be measure with web-analytic data that is internally collected within the Aim2Be app for children. Adherence will be first operationalized as time spent using Aim2Be, number of days using Aim2Be, and number of tasks completed. Given that web-analytic data tracks all features that children are using and the content utilized, if variability in the data allow it, profiles of users will be used as outcomes.

Mediators that will be examined with respect to adherence are linked to the theoretical framework of Aim2Be (see Fig. 2). The list of mediators are included in Appendix 1 and include 1) an assessment of satisfaction with Aim2Be for gamification mediators; 2) measures of motivation, self-efficacy, and psychological autonomy for behavioral mediators; and 3) assessments of the household environment (family communication and functioning), parenting styles and practices, access/opportunities (cell phone, TV in bedroom, and co-participation in physical activity), modelling healthy behaviors (e.g., parent’s own behaviors), and parental support for environmental mediators.

Moderators that will be examined will be taken from the demographic questions (i.e., age and gender), as well as assessment of enjoyment and preferences for engaging in the behaviors targeted by Aim2Be (five items assessing food and sedentary behaviors preferences and enjoyment for physical activity), knowledge of current health recommendations (eight Yes/No items asking about the physical activity, diet, screen time, and sleep recommendations), weight concerns (two items), self-esteem/optimism (six items) [63], and anxiety/worries (three items) [64].

### Statistical analyses

To address the primary aims, multilevel mixed-effect linear and logistic models, which account for the repeated nature of the data, will be used to test whether zBMI, dietary quality, fruit and vegetable intake, sugar-sweetened beverage intake, physical activity, and screen time at 3 months differ between Aim2Be and control condition participants. To address the secondary aim that examines whether health coaching influences adherence to the Aim2Be intervention, linear regressions will be used to compare the time spent using Aim2Be, number of days using Aim2Be, and number of tasks completed. Three analyses will be run, one for each adherence variables (i.e., time spent in app, numbers of days in app, and tasks completed). As the data are expected to be skewed, the dependent variables will be log-transformed or optimal transformation will be used to deal with the skewness of the data. Finally, examination of the mediators and moderators of adherence will use path analyses and relevant socio-demographic covariates and explore whether recruitment routes influenced adherence (e.g., self-referral or healthcare facility referral). Multiple imputation techniques will be used to deal with the missing data. Analyses will be conducted in Stata (StataCorp LLC, College Station, TX, USA) and MPlus (Muthen & Muthen, Los Angeles, CA, USA).

### Quality assurance and monitoring

Operating procedures will be documented in a Standard Operating Procedure manual to standardize the administration of trial conditions, data collection methods, tracking procedures, and checking programming into REDCap (e.g., randomization and administration of tools within specified timeline). Any adverse events will be reported to both the Childhood Obesity Foundation and the University of British Columbia Children’s and Women’s Research Ethics Board and accordingly added to the trial registry.

### Post-trial care

Study participants will be provided access to the app and any updates to the app for as long as the app is supported by the providers. At the end of the evaluation period, participants will receive emails and a push notification alerting them that the study is complete, at which time they will need to sign a new agreement with the providers if they wish to continue using the Aim2Be app. If a user does not agree to continue using the app, their user account will be deactivated, and a reactivation code will be emailed to them. If they choose to return to the app, they will need to agree to the provider’s agreement. Participants are given the option to continue using the app after the study ends, as there are ethical concerns associated with discontinuing access once the evaluation window is over, particularly if the participant found the program useful.

### Data confidentiality

All questionnaire data will be collected in REDCap hosted at BCCHRI. REDCap is a secure, web-based software platform designed to support data capture for research studies, providing 1) an intuitive interface for validated data capture, 2) audit trails for tracking data manipulation and export procedures, 3) automated export procedures for seamless data downloads to common statistical packages, and 4) procedures for data integration and interoperability with external sources [28, 29]. Personal identifiable information about participants will be kept separate from the main dataset and will not be shared. The main dataset will be deidentified. All data will be stored securely on site at BCCHRI, University of British Columbia, with access limited to authorized study personnel.

### Reporting

Study results will be reported according to CONSORT guidelines. Study findings will be submitted to a peer-reviewed scientific journal for publication and be presented at academic conferences. The study results will also be reported in the ClinicalTrials.gov registry.

## Discussion

Although in-person family-based interventions are the standard for supporting families of children with overweight or obesity, these programs are costly and may not reach all those who can benefits from such programs. In addition, these programs often do not meet the needs of many families [12–16]. Therefore, mHealth interventions may provide a viable alternative to in-person family-based interventions; however, the efficacy of such interventions have not been evaluated robustly in the current literature. Evidence from the current RCT will strengthen the current mHealth literature. This RCT will examine the efficacy of Aim2Be in decreasing zBMI scores and improving lifestyle behavior factors. Beyond this, the RCT will examine whether Aim2Be can provide a practical and accessible intervention that improves adherence to lifestyle interventions.

One limitation to the study is the short intervention period, which may not allow for sufficient time to observe changes in the BMI z-score, particularly in an adolescent population. However, the co-primary outcome data on lifestyle behavior (physical activity and diet quality) may provide greater insight into the overall efficacy of Aim2Be. Furthermore, the mediators of behavior change and predictors of adherence will provide insights as to whether the intervention worked as intended. As the trial has a number of inclusion and exclusion criteria, we will gain a better understanding of who is reached by these mHealth lifestyle interventions in these criteria and for whom it appeals to the most. Gaining a better understanding of the mediators and moderators that are linked with intervention adherence will serve to advance the understanding of how mHealth interventions work.

## Trial status

The trial is scheduled to be completed by November 30, 2020. Recruitment began on January 2, 2019, and will be completed by approximately October 31, 2019.

## Supplementary information

**Additional file 1.** SPIRIT Checklist.

**Additional file 2.** Consent Form.

## Data Availability

Please contact Dr. Louise C. Mâsse at the BC Children’s Hospital Research Institute/School of Population and Public Health University of British Columbia (lmasse@bcchr.ubc.ca) regarding access to data and materials.

## Abbreviations

ACUDO: Agency, Challenge, Uncertainty, Discovery, Outcomes
BCCHRI: British Columbia Children’s Hospital Research Institute
BCT: behavior change techniques
BMI: body mass index
CHEO: Children’s Hospital of Eastern Ontario
LIGHT: Living Green Healthy and Thrifty
PENS: Player Experience and Need Satisfaction
RCT: randomized controlled trial
SCT: Social Cognitive Theory
SDT: Social Development Theory
SPIRIT: Standard Protocol Items: Recommendations for Interventional Trials
WEBQ: Waterloo Eating Behavior Questionnaire
