# Supplementary material for: Aim2Be mHealth intervention for children with overweight and obesity: study protocol for a randomized controlled trial
Source: Trials. 2020 Feb 3;21:132. doi: 10.1186/s13063-020-4080-2 (PMC6998843; doi:10.1186/s13063-020-4080-2)
Supplement: Supplementary file 2 — Additional file 2. Consent Form. [file 13063_2020_4080_MOESM2_ESM.pdf]

PARENT CONSENT FORM
Living Green and Healthy for Teens (LiGHT)
Phase 4

Sponsor
Development and evaluation of the app is funded by the Public Health Agency of Canada (PHAC), Ayogo Health Inc., Merck Canada Inc., Heart and Stroke, Diabetes Canada, Obesity Canada, Craving Change, the David Suzuki Foundation, and Pacific Blue Cross. Evaluation of predictors of adherence to the app is sponsored by Canadian Institutes of Health Research, Alberta Innovates, Canadian Obesity Network, Women and Children's Health Research Institute at the University of Alberta, and the Ontario Ministry of Health and Long Term Care. Following this evaluation, it is intended that the program be available in Canada free-of-charge; however Ayogo Health Inc. plans on commercializing the program in other countries. The University of British Columbia, the Childhood Obesity Foundation, and Ayogo Health Inc. all have intellectual property interests in the program.

Purpose
This evaluation is conducted by the University of British Columbia (UBC) in collaboration with the Childhood Obesity Foundation and Ayogo Health Inc. Living Green and Healthy for Teens (LiGHT) is a project that seeks to learn more about Aim2Be, a mobile app that aims to help young people and their families develop healthy eating habits and active lifestyles. The evaluation intends to determine: 1) how well Aim2Be works; 2) whether the strategies we use to support change in children's health behaviours are helpful; and 3) whether the content of the program is engaging.

Procedures
About 200 children and their parents will enroll in this study. You and your child will be given unlimited access to Aim2Be for three or six months, and asked to use the program according to its instructions. You will be randomly assigned to one of two groups: 1) access to Aim2Be v2.2 with a live coach as well as a virtual coach; or 2) access to Aim2Be v2.2 with a virtual coach after a 3-month waiting period.

You and your child will be asked to each complete online questionnaires at the start of the program, after three months and after six months. These questionnaires will ask questions about how well the program functioned and whether you enjoyed it, demographic characteristics about your family, your knowledge of Canadian recommendations for healthy eating and physical activity, some of your lifestyle behaviors, and motivation and household/family characteristics. At three times during the study period, your child will also participate in health behavior assessments. You will be provided with a Fitbit (a physical activity monitor), a scale, and a measuring tape when you enroll in the study. The activity monitor is a small, unobtrusive band that is worn on your child's wrist continuously for the two-week period. At each assessment date your child will be asked to wear it for two weeks and report their height and weight. They will also be asked to recall everything they ate during the past 24 hours for three separate days. These assessments can be done from your home, but your child may require your assistance.

When using the app, a web-analytic tool will track how much of the content you view, how often you log in, and how well you adhere to the goals you set. It will not track any personal information or track anything you do outside of the program. All of this information will be kept confidential.

After the study is completed, you will be able to continue using the app if you choose.

Using the App
If you use the app, you and your child must adhere to Aim2Be's Community Guidelines. These guidelines ensure that you treat all users in the app with respect. If you or your child do not comply with these Community Guidelines, you may be excluded from the study and your access to the app may be restricted.

The app contains information relating to health topics, such as healthy eating, active living, sleep, and screen time, for educational purposes only. Always seek the advice of your doctor or other healthcare professional with any questions you may have about medical symptoms or a medical condition. The app is not meant to replace your existing healthcare providers, such as your family doctor.

Ayogo Health Inc. and the Childhood Obesity Foundation hold copyright on all intellectual property related to the app, software and the content. You may only use the app for personal use and the stated purposes of the study.

In order to download the app through Google Play or the App Store, or by viewing it in a web browser, you will be using software from third parties that are not associated with this study. These third parties may ask you to agree to their terms and conditions.

You and your child may grant the app access to your phone's camera and Fitbit step counter, if you wish. If you grant access to your camera, you and your child may post photos to the app's Social Wall, in order to document completion of certain healthy behaviours. These photos will be viewable by other users. Any photos you post to the app that contain faces or other identifying features will be immediately removed.

After the Study
You will be provided access to the app, and any updates to the app, for as long as you wish. You will receive up to three emails and a notification on your device when the study is completed. <u>If you wish to continue using the app at this time, you must agree to Ayogo's End User License Agreement (EULA)/privacy policy. You may also delete your user account at any other time before or after the study ends. If you choose to delete your user account or do not agree to the EULA/privacy policy at the end of the study period, your email address will be deleted from Ayogo Health Inc's servers. Any information that you have already provided up to that point will not contain any identifiers and will be used only for analysis purposes and to improve the app</u>. Any comments you have made on the social forum will have your username removed, but the comment itself will still be visible to other users.

<u>If you agree to continue using the app at the end of the study period, your encrypted email address will remain on Ayogo Health Inc's servers, information you have already provided may continue be used by the</u>

<u>app to personalize it to your preferences, and Ayogo may continue to collect data on how and when you use the app.</u>

If you do not continue using the app at the end of the study period, you will be provided with a reactivation code. If at any time you wish you reactivate your user account, you may enter this code and your access to the app will be restored, after agreeing to Ayogo's EULA/privacy policy and providing your email address.

Results
The results of this study will be used to inform changes to future versions of Aim2Be. They may also be published in academic journal articles.

Study Participant Criteria
In order to participate, you must meet the following criteria:

- Your child is between 10 and 17 years old
- You and your child are literate in English and are able to read at the grade 5 level or above
- You have a computer or mobile device, and internet access at home
- Your child primarily lives in your household
- Your child has a body mass index that classifies them as overweight or obese

Potential Risks
There is a possible – but unlikely – risk that participants may experience injury or illness as a result of increasing physical activity or making recommended dietary changes in the program. There is also a small, but unlikely risk that wearing the Fitbit for prolonged periods may cause skin irritation. The Fitbit also contains electrical equipment that could cause injury if not handled properly. If your child feels soreness, tingling, numbness, burning, or stiffness in their hands or wrists while or after wearing the product, discontinue use and inform the researchers of this as soon as possible. Some of the topics that are explored in the program deal with factors that prevent your family from having a healthier lifestyle, and how your child views themselves. Some of these topics may seem sensitive or raise feelings that might upset you or your child. Some of the questions we ask may seem sensitive or personal. You do not have to answer any questions if you do not want to. If you or your child disclose risk of harm, researchers will report it to the Ministry of Children and Family Development or contact emergency services if immediate.

Potential Benefits
By participating in this evaluation, you will have the opportunity to learn techniques and behaviours to improve you and your family's health and save money. By participating in this evaluation you will also be helping to make the program better for other Canadian families.

Confidentiality
All data will be identified only by a code number and will be kept on secure password protected webservers, which will only be accessed by the study staff from UBC, Ayogo Health Inc., and the Childhood Obesity Foundation. Your name and contact information will be used by the Childhood Obesity Foundation so that you can be contacted to receive support from a live coach. Ayogo Health Inc. will have access to your email address in order to register you for the Aim2Be app and send you reminders to use the program.

Your contact information will never be shared with anyone outside of UBC, Ayogo Health Inc., or the Childhood Obesity Foundation and will only be used for the purpose of conducting this evaluation. Anyone who works with your data will sign a confidentiality agreement. The majority of the evaluation will be administered using an online database program called REDCap and through the Aim2Be platform. All information collected through REDCap will be stored on a private, relational MySQL database at the data centre which is located on-site at BC Children's Hospital in Vancouver, BC. The data stored in REDCap will contain your first name and email address, but no other identifying information. All other data will not be attached to your personal information, and identifying information will be stored separately. Identifying information will be collected using a separate REDCap database. All information collected through the Aim2Be platform will be stored on the Ayogo Health Inc secure server and will be transferred electronically to the UBC server using encryption software. All data transfers to UBC will occur using a secure connection to maintain the confidentiality of your data at all times and no identifiers will be included in any data that is transferred from Ayogo Health Inc to UBC. The dietary habits questionnaire will be administered through the University of Waterloo and your child's activity tracking (Fitbit) data will be collected by Fitabase. All of this data will only be identifiable by a code number, and neither the University of Waterloo or Fitabase will have access to your personal information. All data we collect, except Fitbit data, will be stored in Canada. Research staff will authorize Fitabase to access and download your child's Fitbit data for the duration of this study. Fitabase is a fully hosted, cloud-software solution that implements robust industry standards to maintain secure databases and it was specifically developed to support research like this. As Fitabase stores data in the US, all data collected will be anonymous and a de-identified study ID number will be used within the Fitabase platform, so Fitabase will have no information about your child. Research staff from UBC will be able to access, view, and download the Fitbit data through the Fitabase platform.

The information you provide will only be used for the purposes of this evaluation and for administering and improving the app. The data will be kept by UBC for a minimum of five years after publication of results. De-identified data will be kept by Ayogo indefinitely, as they will be used to inform ongoing development of the app. Any reports of the completed study will present only grouped data so that you will never be identifiable. All documents, except your consent form, will identify you only by your code number. All paper documents will be kept in locked file cabinets.

If you participate in the social forum, anything that you share can be viewed by other users. We encourage all participants who use the social forum to refrain from sharing any content that other users post; however, we cannot control what other participants do with the information they view. Users are encouraged to use good judgement about what they post, and be careful not share any personal information. The social forum will be moderated, and personal information or inappropriate content will be deleted. If posts on the social forum or conversations with the live coach indicate that you or your child are in distress or crisis, you may be contacted by a live coach and provided with resources. Conversations with the live coach will be kept confidential unless the live coach has reason to believe that you or someone else may be harmed. Any information that would indicate that a child was being harmed or at risk of such harm, will not be kept confidential and instead be disclosed as legally required to the appropriate authorities such as the Ministry of Children and Family Development in cases of suspected abuse or neglect, or emergency services if at immediate risk of harm.

Linked Data Use

Data collected in this study will also be used in a separate study being performed by Dr. Louise Mâsse, that will evaluate whether participants used the app more if they had the virtual coach or both the live coach and virtual coach, and what factors influence participant's ongoing use of the app. This data will not be shared with anyone outside of Dr. Mâsse's research team at UBC.

Payment

Your family will receive $60 for participating in the baseline assessment, an additional $80 for participating in the three-month assessment, and an additional $100 for participating in the six-month assessment. This amount will be prorated if any of the questionnaires have not been answered by either you or your child. You will receive payment via e-transfer, at the email address you provided when you enrolled in the study. If you do not wish to receive payment via e-transfer, you have the option of receiving the payment via cheque. You will also get to keep the scale, measuring tape, and Fitbit that are mailed to you for the study.

Contact for Information about the Evaluation

If you have any questions or desire further information with respect to this evaluation, you may contact a member of our staff at 604-875-2000 ext: 6393.

Contact for Concerns about the Right of Research Subjects:

If you have any concerns or complaints about your rights as a research participant and/or your experiences while participating in this evaluation, contact the Research Participant Complaint Line in the UBC Office of Research Ethics at 604-822-8598 or if long distance email RSIL@ors.ubc.ca or call toll free 1-877-822-8598.

Consent to Participate

Taking part in this study is entirely up to you. You and your child have the right to refuse to participate in this evaluation. If you decide to take part, you may choose to pull out of the study at any time without giving a reason and without any negative impact on you or your child access to care in any way. If you require more time to consider your participation, you may come back to this page and complete this form at a later time.

By providing consent, you are indicating the following:

- You have read and understood the evaluation information and consent form.
- You have had sufficient time to consider the information provided and to ask for advice if necessary.
- You understand that you will receive a copy of this consent form and your child's assent form for your own records

---

□ I consent to participate and consent to my son/daughter's participation in this study

□ I consent to be contacted at a later date to participate in other research activities related to the LiGHT program

□ I understand that I will be notified when the study period is completed and that if I agree to continue using the app at that time, my email address will remain on Ayogo Health Inc's servers. If I do not agree to continue using the app, then my user account and my email address will be deleted.

ADOLESCENT ASSENT FORM

Living Green and Healthy for Teens (LiGHT)

Phase 4

I am being invited to take part in this research because I want to learn how to make healthier choices about food and exercise. This form explains the study to me so that I can decide if I want to take part or not. It is up to me if I want to be in this study. No one will make me be a part of the study and no one will get mad at me if I don't want to be a part of it.

What is the study about?

This study is called Living Green and Healthy for Teens (LiGHT). The LiGHT study hopes to learn more about Aim2Be, a cell phone app that will help me and my family learn how to stay active and eat well. I am being invited to be in this study because the researchers want to find out what I like and don't like about the app, how I am using it, and how the app changes my activities, my health, and my knowledge. My feedback will help the researchers to make the app better for other people to use. In total, up to 200 families will be participating in this study.

What will I do in this study?

If I decide to take part in the study, I will be asked to use Aim2Be on my phone or computer for three or six months. I will take part in online activities that will help me learn about how to stay active and eat well, and I will be able to talk with a coach, who will help me make healthy changes in my life. The program will also let me talk online to other kids and teens who are trying to make the same healthy changes.

If I decide to take part in the study, the researchers will randomly decide whether I will be put into a group that uses a live coach and a virtual coach, or a group that uses just a virtual coach. If I am put into the first group, I will be asked to use Aim2Be for six months. If I am put into the second group, I will wait for three months, then be asked to use Aim2Be for three months. I will be asked to log in to Aim2Be on my phone or computer a couple of times each week, and complete some activities and learning exercises. This will take about one hour per week.

Regardless of what group I'm in, I will be asked to fill out a questionnaire at the beginning, halfway through, and at the end of the 6-month period. These questionnaires will ask me about my activities, what I've learned from the program, and whether I like using it. These questions will take approximately 1.5 hours to complete in total.

Information about my health and lifestyle will also be recorded at three different times during the study. At each of these assessments, I will be asked to wear a Fitbit that will keep track of how much I move during two weeks. The band is small and does not hurt to wear. At these same times, I will also use an online program to try to remember everything that I ate during the past day. With the help of my parents, I will also weigh myself and measure my height using a measuring tape. These assessments will take about 2 hours, in total. A Fitbit, scale, and measuring tape will be provided to me. The developer will also be able to track how much I am using the program, but they will not be able to see anything private thing I say on my computer. Depending on

the group I am put in, this study could take approximately 15.5 hours or approximately 27.5 hours of my time. This includes all of the time spend using the app on my phone over six months (approximately 1 hour per week).

What will my parents be doing in this study?
One of my parents will also be asked to use Aim2Be, but they will use a special version meant for just parents. One of my parents will answer questions about what they think about the program as well.

What do I need to know about the app?
If I use the app, I must follow Aim2Be's Community Guidelines. These guidelines make sure that I treat everyone in the app with respect. If I don't follow the guidelines, I may not be able to stay in the study.

The app contains information about health, but it is not a replacement for talking to a doctor. I should talk to my doctor if I'm concerned about my health.

I will be given the option to allow the app to access to my phone's camera and Fitbit app step counter. If I give the app access to my camera, I may post photos to the app's Social Wall and these will be viewable by other users. Any photos I post to the app cannot contain faces or other identifying features. If I do post photos that can identify me/other people, they will be immediately removed.

Who can participate in this study?
I can participate if I am between the ages of 10 and 17. I also must weigh more than doctors think is healthy for my age and height. One of my parents must volunteer to be in the study with me and I need to have access to the internet at home. I cannot have any health problems that limit my ability to be physically active, to eat a normal diet, or to function normally at school.

Does my parent need to participate as well?
Yes, one of my parents will need to participate in the study in order for me to participate as well.

Can anything bad happen to me?
It probably will not happen, but I may get hurt or sick if I start being more active or eating different food. There is also a small, but unlikely risk that wearing the Fitbit for too long might irritate my skin. There are also electric pieces with the Fitbit that may hurt me if not used properly. If my wrists hurt while using the Fitbit, I will take it off and tell my parents that it has been bothering me. They will let the researchers know. I may also feel uncomfortable learning about things that will make me healthier and thinking about how I view myself. Some of the questions I will be asked may seem sensitive or personal. I do not have to answer any questions if I do not want to. If I'm ever feeling like I need more help, I can talk to a live coach. If anything I post to the social wall or say to the live coach makes the live coach concerned for my safety, my parents may be contacted. If the live coach thinks I might be harmed, they may also contact other people who can help, such as emergency services or the Ministry of Children and Family Development.

Who will know I am in this study?
Only people who are conducting the study will know I am in it. When the study is finished, the researchers will write a report about what was learned. This report will not say my name or that I was in this study. My parents and I do not have to tell anyone I am in the study if I don't want to.

When do I have to decide?
I have as much time as I want to decide to be part of this study. I am also asked to discuss my decision with my parents.

Is my participation necessary?
It is up to me to decide if I want to be in this study. No one will make me be a part of this study. Even if I agree now to be part of this study, I can change my mind later. No one will be mad at me if I choose not to be a part of this study.

Do I get anything for participating in this study?
My family will receive $60 for participating in the baseline assessment, $80 for participating in the three-month assessment, and $100 for participating in the six-month assessment. We will also get to keep the scale, measuring tape, and Fitbit that are mailed to us for this study.

How will my privacy be maintained?
I will be assigned a code in the study database. My full name and code number will not be put together with my data; however the study database will contain my email address and first name so that the researchers are able to send me questionnaires. All documents will use the code assigned to me so my name will not appear in any reports. Researchers from Ayogo Health Inc. and the Childhood Obesity Foundation will have access to my contact information, but only so they can contact me for reasons related to the LiGHT program. Some information on my diet and activity levels will also be collected by Fitabase and the University of Waterloo, but they won't be able to see my name or any identifying information. This information will be quickly and securely transferred to UBC. My paper data will be kept in a locked filing cabinet. All data will be put on a secure web server which can only be accessed by using a password. Only those who have permission will access the server. All data will be erased/deleted five years after the last study results have been published.

If anything I post to the social wall or say to the live coach makes the live coach concerned for my safety, my parents may be contacted. If the live coach thinks that I might be harmed, they may also contact other people who can help, such as emergency services or the Ministry of Children and Family Development.

Who is doing the study?
Dr. Louise Mâsse from the University of British Columbia, the Childhood Obesity Foundation, and Ayogo Health Inc are doing this study. If I have any questions I can call Dr. Mâsse at 604-875-2000 ext: 5563.

Who do I contact if I have any questions about the study?
If I have any more concerns or complaints about participating in this study, I should contact the Research Participant Complaint Line at the University of British Columbia Office of Research Ethics by email at RSIL@ors.ubc.ca or by phone at 604-822-8598 (toll free 1-877-822-8598).

Assent to Participate

☐ I agree to participate in this study
